# Youth washing as a corporate social responsibility tactic of health-harming industries

**DOI:** 10.1093/heapro/daag018

**Published:** 2026-03-14

**Authors:** Sally Witchalls, Hannah Pitt, Simone McCarthy, Becky Freeman, Samantha Thomas

**Affiliations:** Institute for Health Transformation, Faculty of Health, Deakin University, 1 Gheringhap St, Geelong 3220, Victoria, Australia; Institute for Health Transformation, Faculty of Health, Deakin University, 1 Gheringhap St, Geelong 3220, Victoria, Australia; Institute for Health Transformation, Faculty of Health, Deakin University, 1 Gheringhap St, Geelong 3220, Victoria, Australia; School of Public Health, Level 6, Charles Perkins Centre, Faculty of Medicine and Health, University of Sydney, Sydney 2006, Australia; Institute for Health Transformation, Faculty of Health, Deakin University, 1 Gheringhap St, Geelong 3220, Victoria, Australia; Curtin School of Population Health, Curtin University, Building 609, 7 Parker Place, Bentley 6102 Western Australia, Australia

**Keywords:** children and young people, youth, commercial determinants of health, harmful industries, corporate social responsibility, youth washing, marketing

## Abstract

The marketing tactics of health-harming industries are broader than advertising. Health-harming industries use a range of promotional and public relations strategies to increase corporate branding, promote their products, and shape their image as socially responsible corporate citizens. Increasingly, this has included ‘washing’ strategies which companies use to enhance their legitimacy and deflect blame. There is growing research examining the use of youth-focused engagement in CSR strategies; however, there have been few attempts to document the range of ‘youth washing’ strategies that may be used by health-harming industries. This perspective piece explores some of the strategies that are used across a variety of health-harming industries, including the alcohol, gambling, ultra-processed food, tobacco, and fossil fuel industries, to align with youth activities and causes. We propose a definition of ‘youth washing’ and present some examples of youth washing strategies across five domains—youth education programs; youth employment and training programs; sports activities; youth events; and youth-focused philanthropy. Using this information, we constructed a proposed typology of strategies that will help guide future research and policy considerations of a range of public relations strategies that health-harming industries may use to align their business practices with youth. Academic and policy responses should apply youth-centred approaches to critically examine and counter such practices.

Contribution to Health Promotion

- Introduces and redefines youth washing as a distinct CSR strategy used to enhance legitimacy while deflecting scrutiny.
- Highlights ethical concerns around health-harming industry engagement with youth, particularly in settings that shape health and well-being.
- Constructs a typology of a range of youth washing strategies across education, sport, employment, arts, events, and philanthropy.
- Calls for expanded research and policy attention to youth washing as a commercial determinant of health.
- Advocates for youth-centred approaches to critically examine and counter the narratives and practices of health-harming industries.

## Youth and the commercial determinants of health

The commercial determinants of health (CDoH) can be defined broadly as ‘the systems, practices, and pathways through which commercial actors drive or negate health and equity’ (Gilmore *et al*. 2023, p. 1195). While corporations and commercial entities can have a positive impact on human and planetary health and equity (Douglas *et al*. 2024), most research in the CDoH has focused on health-harming industries such as tobacco, alcohol, gambling, ultra-processed foods, and fossil fuels, which have caused wide-reaching harm to human and planetary health (Gilmore *et al*. 2023). Commercial actors contribute to harm in both overt and hidden ways, with some playing significant roles in developing health-harming industry strategies known collectively as the needed in front of ‘corporate playbook’ (Lacy-Nichols *et al*. 2022, Thomas *et al*. 2024). These corporate playbook strategies can range from the development of ‘healthier’ products that may still pose harms to health (Collins *et al*. 2019), public relations and framing (Weishaar *et al*. 2016), disrupting science (Raynes-Greenow *et al*. 2021), and commercial marketing strategies (Westling *et al*. 2025). These strategies are used to promote the consumption of products, create positive attitudes towards corporations, and resist policy reforms (Ngqangashe *et al*. 2024). The World Health Organization (WHO) acknowledges that when commercial actors are able to avoid the costs that arise from products that harm health and equity, they undermine public health (WHO 2025b). Gaps in strong government regulation have enabled health-harming industries to use these strategies to evade accountability, allowing further health and planetary harms to continue (WHO 2025b).

As with the social determinants of health, the CDoH can exacerbate health and social vulnerabilities for some population subgroups (Thomas *et al*. 2024). Youth (defined by the WHO as those 10–24 years old) (WHO 2025a) are at increased risk of negative outcomes from the tactics and strategies of a range of corporations (Pitt *et al*. 2024b). Industries with products and practices that can harm the health of youth include tobacco and vaping (Dennison *et al*. 2021, McCausland *et al*. 2024), alcohol (de Goede *et al*. 2021), gambling (Moodie *et al*. 2024, Pitt *et al*. 2024b), ultra-processed foods (Khoury *et al*. 2024), and fossil fuels (Perera and Nadeau 2022). These harms can be exacerbated for youth who may already be at risk of health and social inequities, due to other determinants of health that they experience (Schrecker *et al*. 2018, Wood *et al*. 2023, Rad 2025).

To date, much of the existing research examining the impact of health-harming industry tactics on the health and well-being of youth has focused on commercial advertising (Boelsen-Robinson *et al*. 2016, Packer *et al*. 2022, Soraghan *et al*. 2023, Pitt *et al*. 2024b, Worters *et al*. 2025). However, marketing tactics are much broader than advertising. Health-harming industries use a broad range of promotional and public relations strategies to increase corporate branding, promote their products, and shape their image as socially responsible corporate citizens, including direct and indirect advertising, promotion, sponsorship, incentives, public relations, lobbying, and charitable donations (Gilmore *et al*. 2023, Thomas *et al*. 2023, p. 3). While a focus on the impact of advertising remains important, limited research has examined how this broader range of public relations strategies may engage and influence youth and their parents and carers (van Schalkwyk *et al*. 2022b, McCarthy *et al*. 2024a, Pitt *et al*. 2024a).

## The use of corporate social responsibility strategies by health-harming industries

Researchers have identified the range of public relations strategies that some industries use to influence, engage and build relationships with key stakeholders, avoid policy reform, and soften perceptions of risk through efforts to positively shape public attitudes (Mialon and McCambridge 2018, McCarthy *et al*. 2024a, Ngqangashe *et al*. 2024). While public relations strategies are often excluded from conceptual frameworks that seek to explore the impact of corporate tactics on health (Maani *et al*. 2020), CDoH researchers have started to investigate how corporate social responsibility (CSR) strategies are used by health-harming industries (McCarthy *et al*. 2024a, Amul 2025, Freeman *et al*. 2025, Murray *et al*. 2025, Brugman *et al*. 2026). From a business perspective, CSR strategies are framed as a range of ‘social good’ initiatives that businesses can engage in to demonstrate their environmental, ethical, philanthropic, and economic responsibilities to communities (Carroll 2016). CSR may also be used as a form of self-regulation, so government intervention and regulation are perceived as not being needed (Palazzo and Richter 2005), as well as a powerful marketing tool that can help a company position itself favourably in the eyes of consumers, investors, and regulators (Ponte *et al*. 2009, Stobierski 2021). This is why they are valuable for the reputations of industries that may pose risks to public health.

There has been a particular research focus on the tactics of the alcohol industry (Maani *et al*. 2024, Amul 2025, Bujalski 2025), with studies demonstrating how CSR strategies are used by the alcohol industry as a tactic to frame community and policy discussions and promote ‘moral agency’ (Gómez *et al*. 2024, p. 5). Framing is important as it influences how individuals understand and react to issues (Maani *et al*. 2022). For example, Yoon and Lam (2013) argue that the alcohol industry uses CSR strategies to create an ‘illusion of righteousness’ (p. 1) through philanthropic sponsorship and promoting strategies that support voluntary self-regulation. In a systematic review of CSR strategies by the alcohol industry, Mialon and McCambridge (2018) identified five strategies used to frame the nature of alcohol-related issues in line with health-harming industry interests, including alcohol information and education provision; drink driving prevention; research involvement; policy involvement; and the creation of social aspect organizations. Similar strategies have been used by the tobacco industry globally, including implementing youth smoking prevention programs, providing financial support to Non Government Organisations (NGO), creating voluntary marketing codes (McDaniel *et al*. 2016), developing so-called ‘healthier’ products and using health professionals to add credibility to these products (Hendlin *et al*. 2024).

Researchers have started to examine the impact of CSR strategies on youth. For example, researchers have demonstrated that ‘harm reduction’ strategies promoted by the tobacco industry created confusion about the health risks of smoking for youth (Fitzpatrick *et al*. 2022, Watts *et al*. 2024, Rose *et al*. 2025). An analysis of Philip Morris Internationals’ (PMI) Smoke Free Future campaign found that youth were unable to identify the tobacco industry as the message source, instead seeing the campaign as genuinely supportive of smoke-free initiatives, and expressed interest in smoke-free products (Evans-Reeves *et al*. 2021). This CSR strategy implemented by PMI seeks to shift the focus from cigarettes, its most profitable and popular product, to its other suite of claimed ‘healthier’ nicotine products (Fitzpatrick *et al*. 2022). Researchers have also shown that youth hold positive views about the CSR strategies of health-harming industries, which they perceive can offset the negative impacts of these health-harming industries (McCarthy *et al*. 2024a).

## The development of ‘washing’ strategies as a specific form of CSR

Health-harming industries use a range of ‘washing’ strategies, to encourage more favourable public narratives about companies and their practices (Gatti *et al*. 2019, Sobande 2020). Examples of ‘washing’ include: *green washing*—making claims that products are less harmful for the environment than they actually are (Gatti *et al*. 2019, Montiel 2020, Fella and Bausa 2024); *sports washing*—promoting sport to redirect public attention away from unethical conduct (Hayhurst 2011, Batty *et al*. 2016, Bergkvist and Skeiseid 2024, Irwin 2025); *pride washing*—using performative allyship to associate with LGBTQIA+ communities (Schopper *et al*. 2025), *pink washing*—for breast cancer CSR initiatives (Lubitow and Davis 2011); *gender washing*—presenting as supportive of issues related to gender without making meaningful changes to address gender-based issues (Calkin 2016, Gregoratti 2016, Gerard 2019, Walters 2022, 2024, Jester and Walters 2024); and *health washing*—using inappropriate and misleading claims about the health benefits of products (Collins *et al*. 2019, Heiss *et al*. 2021, Ares *et al*. 2023). Experts including youth advocates have highlighted the increasing use of *youth washing* as a tactic used by health-harming industries, which refers to platforming youth voices in a performative and tokenistic way instead of acting on the concerns of these groups or including them in meaningful discussions about these issues (The Climate Reality Project 2023, UK Youth Climate Coalition 2024, May and McKenzie 2025). These tactics do not always occur in isolation and may intersect with other washing strategies—for example, greenwashing and youth washing may be strategically combined to leverage young peoples’ concerns for the environment (Sawicka and Marcinkowska 2023, Arnot *et al*. 2024).

## The nature and extent of health-harming industry youth washing strategies

Companies may make specific mention of youth in their organizational CSR activity planning documents (Corporate Justice Coalition 2014, Berlan 2016). There has been increased focus on youth washing particularly in relation to climate, defined by youth climate advocates as:

The act of inviting young members of society to meetings with high profile individuals with the aim of appearing to be listening to the younger generation’s voice and creating positive progress (Brittan 2023).

Youth washing strategies employed by industries that are harmful to health can extend across many youth-focused activities, including education, sports, and event and charity settings. While there is growing research examining the positioning of youth in CSR strategies (Morgan *et al*. 2024), very few studies to date have sought to identify the range of youth washing strategies that may be used by health—harming industries in their CSR strategies and tactics. Starting to document the extent and range of some of these activities is important in trying to distinguish the difference between what may be considered ‘genuine engagement’ and youth washing. Without a range of examples, it may be difficult for people to understand why certain activities linking health-harming industries with youth (e.g. educational scholarships or programs) may be problematic from a public health perspective. Some may see these activities as an inherently positive way for these industries to help account for the harms that they cause. Without clear examples, it is difficult to assess any ethical tensions and conflicts of interests in these types of relationships—particularly those that involve industries talking directly to youth and building their support.

This perspective piece describes some of the youth washing strategies that are used across the alcohol, gambling, fossil fuel, tobacco, and ultra-processed food industries. These industries are the focus of this paper because of the documented risks they pose to children and young people. We present a typology of strategies, as well as suggestions for future research and policy action. We draw upon examples that highlight youth washing tactics. These have been collected through a broad exploratory scan of publicly available materials found through online searches of company websites, social media posts, and strategic company plans. We also scanned for examples in published academic literature. The scan was conducted between January and September 2025 and largely focused on recent examples from 2020 to 2025. We aimed to identify examples that, while not exhaustive of all potential youth washing practices, may highlight the variation and complexity of youth-focused CSR initiatives. For the purposes of this paper, we have collected examples based on a proposed expanded definition of youth washing. This definition expands previous definitions (Brittan 2023) to a working definition of:

The strategies used by health-harming industry actors to align themselves with young people or youth development initiatives, that may be used to enhance legitimacy and public image, while diverting attention from practices that may be harmful to youth or invite public scrutiny.

The paper considers the following research questions:

1. Is there evidence that a range of health-harming industries are using strategies that could be classified as youth washing?
2. What is the nature and scope of these strategies?
3. What are the next steps for research to better understand the health impact of youth washing strategies?

## Youth education-focused CSR activities

Some health-harming industries provide financial support for education-focused activities. Researchers have identified that educational settings may be one way of influencing youth (Pulimeno *et al*. 2020), either through public health education (Woodward-Lopez *et al*. 2023, Carvalho and Vilaça 2024, Kontak *et al*. 2025), or through involvement in education that can undermine health goals (Wilkinson 2024). These activities are framed to encourage youth to investigate healthier environments and adopt lifestyle choices, to better their futures (van Schalkwyk *et al*. 2022b). Some of these strategies include in-school programs, scholarships, and educational campaigns.

### In-school programs and youth education campaigns

In-school programs and youth education campaigns funded directly or indirectly (e.g. through charities and front groups) typically provide classroom resources for teachers and support extracurricular activities focused on problem-solving (Coombs *et al*. 2011, van Schalkwyk *et al*. 2022a, Wilkinson 2024). An example of an educational program that focuses on youth is the Science Technology Engineering and Mathematics (STEM) program supported by oil and gas production company Woodside Energy in Western Australia (Woodside Energy 2025). This program has trained Woodside volunteers working with already existing education curriculum and classroom-based learning to deliver Earth science education, including delivering national STEM competitions (Jenkinson *et al*. 2014, Woodside Energy 2025). A component of this work is the Woodside Australian Science Project, which produces:

…support packages for the Earth Science component of the Australian Curriculum… (STEM) learning packages have also been developed to engage students with real problems in earth science and the STEM approach that is required to solve them (Woodside Australian Science Project 2024).

The project uses future framing to describe helping students to understand real world challenges in Earth sciences, for the success of the future:

Our success as a State is highly dependent on increasing our understanding of the sciences, and in particular earth science, as we seek to maximise the opportunities we have in natural resources (Woodside Australian Science Project 2024).

Positioning the company name within the name of the program may normalize these industries in the lives of youth. Other education programs identified included extra-curricular school activities for youth, often linked to solving world issues and working on topics in STEM or health and life skills for future well-being (Andrée and Hansson 2020, van Schalkwyk *et al*. 2023).

Education strategies have also included the creation of educational and harm minimization campaigns and content that youth are exposed to. A core component of these education campaigns is the personal responsibility element of the materials. This is recognized by researchers as an approach used to deflect responsibility from the companies’ practices (Friedman *et al*. 2015, Goltz 2022, van Schalkwyk *et al*. 2022a). For example, researchers have highlighted examples of alcohol industry involvement in funding organizations that focus on lifestyle messaging as a part of their CSR work, including making healthy choices and mitigating the health harms of alcohol consumption (Maani Hessari *et al*. 2019). An example of this is DrinkWise, an Australian organization whose stated aim is to promote generational alcohol consumption trend change, while its work is, ‘funded primarily through voluntary alcohol industry contributions’ (DrinkWise Australia 2024). DrinkWise describes itself as being independent (Cook *et al*. 2025), working through education campaigns and resource creation, in an attempt to create partnerships with stakeholders across academia, government, community organizations, and the alcohol industry (Pietracatella and Brady 2016). DrinkWise introduced the *Never Have I Ever* campaign, stating it was intended ‘to help support young people when it comes to their mental health and healthy coping strategies’ (DrinkWiseAustralia 2023); the campaign was supported by the Australian Hotels Association (a peak body for hotels in Australia which also provide a range of alcohol and gambling products through poker machines and in pubs). The campaign uses visual aids in pubs around New South Wales to encourage youth to think about their own and their loved ones’ mental health, with the campaign recognizing alcohol as a coping mechanism for poor mental health. Studies have investigated the public understanding of DrinkWise, which shows the public is unaware that the alcohol industry funds some of the organizations work (Carah and van Horen 2011, Brennan *et al*. 2017).

### Educational scholarships

Scholarships are another example of youth-focused funding. Scholarships can differ in value and focus, and can be targeted to certain groups of youth, for example, young women interested in mining engineering by fossil fuel company BHP (BHP 2023). Scholarships are at times linked with academic institutions or run independently by companies. Financial assistance from these industries is given for a range of educational support costs, including study fees like the Australian Indigenous Education Foundation tertiary scholarships that are funded by BHP (Australian Indigenous Education Foundation 2024), attendance at summits (One Young World 2024), as well as for professional development opportunities (BHP 2023). An example of an educational scholarship is bp’s (British Petroleum; one of the world’s largest oil and gas companies) Net Zero scholarship, which is offered globally to youth aged 18–30 years (bp 2023). The scholarship provides candidates with travel and accommodation for the One Young World Summit, and bp engagement through briefing sessions with the candidates. It seeks candidates who are making an impact in areas including advocating for progressive climate policies, enhancing current low-carbon energy technologies such as solar and wind, improving access to low-carbon energy and innovating to reduce emissions in households, regions, cities, and industries (One Young World 2024).

… bp is pleased to support the Net Zero Scholarship, a programme to identify and engage with young innovators who are reimaging energy for people and planet today (One Young World 2024).

## Youth-focused employment programs

Employment programs offer students opportunities with a range of companies that fund these programs. Examples include the mining, engineering, or science fields for fossil fuel industry-funded programs, such as the mining employment programs run by Glencore (2025). Graduate programs seek to engage young people, often providing opportunities for those new to the workforce. An example of this is fossil fuel company Shell Australia, which has graduate and employment programs in their community-focused work (Jenkinson *et al*. 2014, Shell Australia 2024). This includes apprenticeship programs for Aboriginal and Torres Strait Islander students.

The Warrmijala Murrgurlayi (Rise Up To Work) program is helping young Indigenous locals in Broome gain work-ready skills for a career in the region. Supported by Shell and delivered by Nyamba Buru Yawuru (Yawuru), the program’s agricultural component is encouraging local Indigenous youth to feel a greater connection to their culture and country (Shell Australia 2025a).

## Sports-focused CSR activities

CSR strategies can also use sport as a vehicle for their PR tactics. Physical activity, and by association organized sport, can engage youth and improve their health outcomes. CSR in sports differs from other sectors such as education and the arts, due to sport’s unique cultural ties to media influence, youth engagement, social dynamics, and ability to advocate for social causes (Zargar and Rynne 2023). Sport has significant positive social and community impacts, which health-harming industries leverage to promote their agendas (Fruh *et al*. 2023, Bergkvist and Skeiseid 2024). Youth washing and sportswashing combine for CSR strategies with strong corporate benefits such as the avoidance of product regulation and preventing the tightening of advertising restrictions (Bergkvist and Skeiseid 2024).

### Youth sports club funding

The corporate image gained from sponsoring local sports clubs is beneficial to health-harming industry brands, and economic involvement from health-harming industry is helpful to local clubs (McCarthy *et al*. 2024b). Local sports clubs and sport-specific organizations have often relied on their funding from health-harming industry sponsorship. This funding can include agreements of logo placement on uniforms and infrastructure, and naming rights of awards for athletes and teams sponsored by the company. Monetary contributions often go towards equipment and activities that are part of running a sports club. There is a strong association between health-harming industry involvement in sport and consumption of the products manufactured by that health-harming industry by the members of the sponsored club (Sullivan *et al*. 2011). The alcohol industry also sponsors sports clubs with junior programs (Gonzalez *et al*. 2020), including Cooper’s Brewery funding of Norwood Australian Rules Football (AFL) Club in South Australia (Norwood FC 2024), which has a U18 and U16’s team.

### Youth sports programs and social impact programs

Similar to health-harming industry funding youth sports clubs, they can also sponsor sports programs that cater towards young people. Across Australia, sports programs are available for those as young as 3-years-old (Little Athletics Australia 2024, Tennis Australia 2025). Some of these programs are sponsored by health-harming industry, with branding rights often part of the funding package. For example, fast food company McDonald’s Australia works with a range of youth sports programs. One program is ‘Macca's on your Team’—where McDonald’s partners with the Australian states Victoria and Tasmania, to fund cricket, basketball, and AFL community leagues, including supporting the governing bodies and providing equipment (McDonald's Australia 2025a). Another program is the ‘Macca’s Junior Sports Grants’ in Western Australia (McDonald's Australia 2025b). McDonald’s Australia also supports Little Athletics, Swimming Queensland, and South Australian National Football League, and their associated sports programs, and supplies achievement awards to community (including sport) groups in the form of restaurant vouchers for local McDonald’s restaurants (McDonald's Australia 2025c). McDonald’s Australia say of their support of these programs:

We like to focus on activities that improve the health and wellbeing of Australian children. We contribute to sporting organisations that develop kids’ sports skills and provide opportunities for them to play. Hundreds of sporting clubs across Australia benefit from the support of their local McDonald’s restaurant and in some states restaurants work together to support state-wide programs (McDonald's Australia 2025c).

This framing by McDonald’s Australia has been criticized as counter-intuitive, given that their food and beverages are largely considered unhealthy and the ultra-processed food industry is well established as negatively impacting the health of youth (Singh *et al*. 2021, Jia *et al*. 2022).

The use of social causes and sport to engage young consumers from a CSR standpoint has been implemented globally by health-harming industries, with social justice issues regularly involving youth. An example of this strategy is the AFL Gender Equity Education Program supported by BHP, which:

… supports community football clubs to develop the knowledge and skills to foster safe, equal and respectful environments for all members of your community, particularly women and girls (Australian Football League 2025).

This program is directly targeted at helping teams attract girls and young women to clubs, by working to tackle issues of gender inequality that may be barriers to participation. Spots for the program are funded by BHP, with the fossil fuel company’s support advertised throughout the program website (Australian Football League 2025).

## Youth event-focused CSR activities

Events provide other avenues for youth engagement. Ranging from recreational activities through to professional development topics, events provide an entry point for health-harming industries to interact directly with young people. Events can intertwine with other youth washing activities identified, such as education, employment, and sport.

### Youth-focused arts events

Health-harming industries leverage arts development events and programs targeting youth as part of their CSR work. These partnerships often align with culturally enriching youth-focused activities, embedding their brand within creative spaces. The Western Australian Youth Orchestra (WAYO) is sponsored by Woodside, including the Side by Side performance, which allows WAYO members to perform with members of the Western Australian Symphony Orchestra (West Australian Symphony Orchestra 2022). BHP is a core education program partner of The AWESOME Festival in Western Australia, whose mission is to provide ever-expanding opportunities for Western Australia’s youth to actively engage with the arts and intensify their connectivity with the broader world in which they live (Barking Gecko 2025). The Texaco Children’s Art Competition is run in Ireland annually, and funded by Texaco, which is an American oil brand, that is currently owned and operated by global energy company Chevron Corporation. The goal of the competition is to support and encourage young people through their art (Valero Energy 2025). Members of the public in Ireland have raised concern with this fossil fuel funding of the competition since at least 2019 (Green News Ireland 2019). All these examples align a health-harming industry to the arts and youth, allowing these companies to frame their CSR work as supporting youth through their engagement with the arts.

### Conferences and competitions

Similar to arts events, conferences and competitions can be sponsored by health-harming industries. Health-harming industries align themselves with events targeted at youth, with health-harming industry sponsorship common at events labelled as youth summits (One Young World 2025, UNICEF Australia 2025). These events often attract young leaders, activists, and entrepreneurs, making them ideal platforms for health-harming industries to showcase their values and products (The Climate Reality Project 2023, UK Youth Climate Coalition 2024). By embedding themselves in spaces that promote youth empowerment and global change, companies can not only gain visibility but also shape narratives around innovation, sustainability, and leadership. Youth-focused competitions are also common events for health-harming industries to be engaged in. Often prizes can be sponsored by health-harming companies, and winners can be promoted in the company’s media plan as positive PR, an example being the Battle of the Minds competition run by the corporate venture capital arm of British American Tobacco (BTV 2023). Events such as science fairs and STEM competitions (Queensland Museum 2025, Shell Australia 2025b) put the responsibility for addressing health and environmental threats onto the shoulders of youth, instead of back on the industries responsible for the harm.

## Philanthropic-focused CSR activities

There are many CSR activities that focus on philanthropic efforts (National Academies of Sciences and Medicine 2016), with funding taking place across sectors such as health, environment, and youth well-being. Research suggests that companies aim to frame their work as helping communities, which has the advantage of helping their product become more desirable for consumers to purchase (Anagnostopoulos *et al*. 2024, Genziuk and Polonsky 2024). Charitable activities for companies can take different forms involving youth, such as forming their own charitable foundation or funding an external charity.

### Charities and foundations

Company-owned charitable foundations use the company name, receive funding from the company, and may report directly into the organization.

An example of this is the BHP Foundation, which is founded and funded by fossil fuel company BHP.

BHP Foundation is a non-profit organization working with others to address the root causes of sustainability challenges relevant to the resources industry. Created and funded by BHP, the Foundation's investments aim to impact the systems that prevent progress towards a more equitable and sustainable future for people and planet (BHP Foundation 2025b).

The BHP Foundation’s work in Australia involving youth includes:

- ‘STEM Together’—Supporting Australia’s future innovation by increasing engagement and participation of underrepresented students in STEM learning and career pathways.
- ‘Right care first time where you live’—Using the latest advances in systems modelling and simulation to empower communities to allocate local resources and funding in youth mental health care in a targeted way.
- ‘Early Years Catalyst’—A national collaboration working with organizations to improve early childhood development outcomes for youth experiencing disadvantage and vulnerability (BHP Foundation 2025a).

Companies can also create and fund partnerships with other well-established charitable organizations. An example of this is Asahi Beverages, which funds charities such as The Smith Family, who help youth in poverty (Asahi Beverages 2025).These foundations highlight their charitable work, often publishing it across traditional and new media platforms, as being beneficial to ‘future generations’, in a PR strategy to redirect scrutiny over health-harming industry practice.

## Youth attitudes towards youth washing

Importantly, research is emerging that explores the range of views that youth themselves have on CSR practices. Those who are critical of CSR strategies were aware of the marketing tactics being used by health-harming industries to push consumption of harmful products (Wang and Juslin 2011, McCarthy *et al*. 2024a). Young people can identify which platforms are more successful in capturing their engagement and have acknowledged that stronger regulation of health-harming industry marketing is needed to safeguard against health harm (McCarthy *et al*. 2024a). In 2019, the UK Youth Climate Coalition started their #stopyouthwashing campaign to raise awareness of the specific use of youth by the fossil fuel industry for PR gain:

Whether it’s physically using young people as the face of their campaigns and promotions, or using narratives which point towards saving the future—it’s hard to argue that fossil fuel companies latest marketing trend is anything other than a blatant attempt to convince the general public that they are in-line with the youth environmental movement (UK Youth Climate Coalition 2024).

This youth washing CSR concern is being identified by youth, who acknowledge they are only being engaged with on a surface level by both health-harming industries, and policymakers (Brown 2021). However, youth also believe that CSR strategies could offset the harm done by health-harming industries (McCarthy *et al*. 2024a). Youth will also make purchase decisions based on perceived social and environmental benefits (Gomes *et al*. 2023). This suggests that health-harming industries are successful in targeting consumers including youth by using tactics that reframe their practices and products as offsetting the harm they cause.

## A youth washing typology

**Figure 1:**
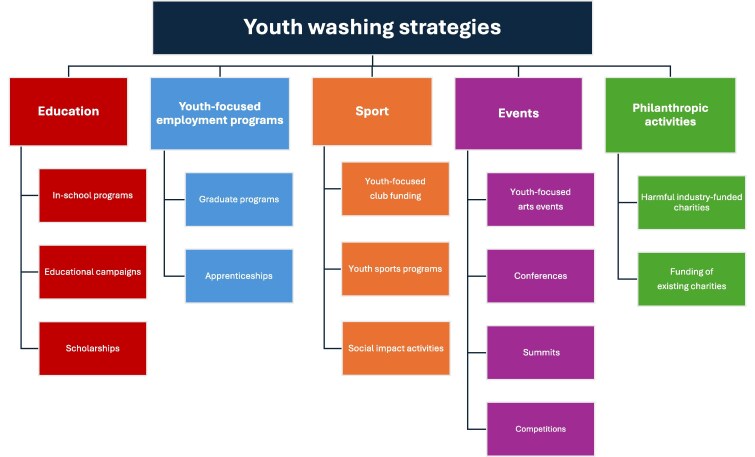
Types of youth washing used by health-harming industries.

The collected examples of CSR practices show that health-harming industries are using tactics to align themselves with youth. These examples exist across various health-harming industries, and work within various sectors such as education, sport, the arts, employment, and events. As a starting point for further exploration related to this issue, we have created a typology based on these examples (Fig. 1). There is the potential for this typology to evolve as new youth washing tactics arise, and further research on youth washing increases globally.

As more examples are identified, our proposed expanded definition of youth washing may need to evolve to better reflect the complexity and intent of practices. This paper also raises questions about whether health-harming industries can ever ethically engage with or engage in activities that benefit youth? Youth washing is not limited to health-harming industries—governments, civil society, and other stakeholders also engage in these practices (Patterson and Linley-Mota 2021, Mueller 2023, Morgan *et al*. 2024). Governments may also form partnerships with industries whose practices may be harmful to health and equity to fund a range of youth activities—including child health research. While this paper focuses on a few prominent industries, there may also be other industries, such as Big Tech, pharmaceutical, and firearms, who may seek to engage youth as a mechanism to prevent or delay regulatory reform. Understanding how a range of stakeholder types use youth washing and for what purpose will help build a more comprehensive framework. This will also help youth, as well as educators, youth workers, and policymakers to identify and critically examine youth-focused CSR tactics and to distinguish these from any genuine forms of youth engagement and support (Berlan 2016, Amoroso and Roman 2019, McCarthy *et al*. 2024a). Further research and stakeholder engagement work is needed to better understand and address the harms of CSR tactics, which will require a multi-stakeholder multi-sector approach centred around youth (Hiswåls *et al*. 2020, Jansen *et al*. 2024). Researchers must talk to youth, endeavour to challenge health-harming industry narratives, and support professionals across all sectors involved with youth, in understanding how youth washing is taking place currently, and advocating for ethical engagement practices.

Talking to youth presents an opportunity for research to move beyond surface-level analysis and begin to develop strategies that challenge youth washing and promote genuine youth engagement and empowerment, including through stronger regulatory levers (Lundy 2007, Arnot *et al*. 2023, Bailey *et al*. 2024, McCarthy *et al*. 2024a, Pitt *et al*. 2024b). Through this initial typology of youth washing practices and expanding the definition of youth washing, we hope to increase discussions and inspire further research on honest, transparent, and youth-rights centred, as per the United Nations Declaration on the Rights of the Child, policy and practices (United Nations General 1959, Lundy 2007). Discussing this work with youth directly will help validate findings and shape more ethical approaches to youth engagement that may lead to a norm of separation with health-harming industries.

## Data Availability

Not applicable
